# Sexual Functioning and Depressive Symptoms in Women with Postpartum Thyroiditis

**DOI:** 10.3390/diagnostics15101286

**Published:** 2025-05-20

**Authors:** Karolina Kowalcze, Gaspare Cucinella, Giuseppe Gullo, Robert Krysiak

**Affiliations:** 1 Department of Pediatrics in Bytom, Faculty of Health Sciences in Katowice, Medical University of Silesia, Stefana Batorego 15, 41-902 Bytom, Poland; 2 Department of Pathophysiology, Faculty of Medicine, Academy of Silesia, Rolna 43, 40-555 Katowice, Poland; 3 Department of Pediatrics, Provincial Hospital in Częstochowa, PCK 7, 42-200 Częstochowa, Poland; 4 Department of Obstetrics and Gynecology, Villa Sofia Cervello Hospital, University of Palermo, 90146 Palermo, Italy; gaspare.cucinella@unipa.it (G.C.); gullogiuseppe@libero.it (G.G.); 5 Internal Medicine and Clinical Pharmacology, Medical University of Silesia, Medyków 18, 40-752 Katowice, Poland; rkrysiak@sum.edu.pl

**Keywords:** depressive symptoms, female sexual functioning, hormonal profile, hypothyroidism, thyroiditis

## Abstract

**Background/Objectives**: The presence of autoimmune thyroiditis was found to be associated with an increased prevalence of sexual dysfunction. Women’s sexual health was not investigated in postpartum disorders of the thyroid gland. The aim of this study was to assess female sexual functioning and depressive symptoms in postpartum thyroiditis. **Methods**: This study compared four groups of non-lactating women who gave birth within 12 months before the beginning of the study: women with postpartum thyroiditis and overt hypothyroidism (group A), women with postpartum thyroiditis and subclinical hypothyroidism (group B), euthyroid females with postpartum thyroiditis (group C) and healthy euthyroid females without thyroid disease (group D). All patients completed questionnaires assessing female sexual function (FSFI), and the presence and severity of depressive symptoms (BDI-II). Moreover, we assessed thyroid peroxidase and thyroglobulin antibodies, as well as serum levels of thyroid-stimulating hormone (TSH), free thyroid hormones, testosterone, dehydroepiandrosterone sulfate (DHEAS), estradiol and prolactin. **Results**: The mean total FSFI score was lower in women with overt hypothyroidism (22.74 ± 4.12) than in the remaining groups of women, lower in groups B (25.71 ± 3.84) and C (29.67 ± 4.00) than in group D (32.15 ± 2.98), as well as lower in group B than in group C. Compared to healthy controls, both groups of women with postpartum thyroiditis and thyroid hypofunction had lower scores for all domains, while euthyroid patients with postpartum thyroiditis had lower scores for sexual desire, sexual arousal and lubrication. The total BDI-II score was highest in group A (15.6 ± 3.2) and lowest in group D (7.8 ± 3.2). Serum testosterone and DHEAS levels were lower while serum prolactin levels were higher in women with postpartum thyroiditis than in healthy subjects. The lowest testosterone levels (1.02 ± 0.35 nmol/L) and estradiol levels (190 ± 80 pmol/L) and the highest prolactin concentration (39.9 ± 13.9 ng/mL) were found in group A. **Conclusions**: The obtained results show that postpartum thyroiditis is complicated by multidimensional impairment of female sexual functioning, which is accompanied by mood deterioration. Severity of sexual dysfunction and depressive symptoms in this clinical entity depends on the degree of thyroid autoimmunity and hypothyroidism. It seems that assessment of sexual functioning and mood should be recommended to all women with postpartum thyroiditis.

## 1. Introduction

Hypothyroidism and autoimmune (Hashimoto’s) thyroiditis, ones of the most frequently encountered endocrine disorders that often affect young women [1,2], were found to exert an unfavorable effect on sexual functioning. Krysiak et al. reported that the mean total Female Sexual Function Index (FSFI) score was lower in euthyroid women with Hashimoto’s thyroiditis and women with non-autoimmune subclinical thyroid hypofunction than in healthy euthyroid females [3]. The unfavorable effects of thyroid hypofunction and thyroid autoimmunity were additive, and women with autoimmune hypothyroidism were characterized by the lowest overall FSFI score and the lowest scores for all domains of the FSFI. Bortun et al. observed that women with autoimmune thyroid disease were characterized by lower FSFI scores than healthy controls, and disturbances in all aspects of female sexual response correlated with the severity of thyroid disease [4]. In the study by Atis et al., female sexual dysfunction (FSD) was diagnosed more frequently in women with overt hypothyroidism (56%) and grade 2 subclinical hypothyroidism (55%) than in grade 1 subclinical thyroid hypofunction (15%) and in age-matched healthy women [5]. The total FSFI score was lower in women with overt and grade 2 subclinical hypothyroidism than in controls [5]. In an Italian study, hypothyroid women but not euthyroid women with Hashimoto’s thyroiditis were characterized by impaired desire, arousal and lubrication in comparison to age-matched women without thyroid disease [6]. Contrary to these findings, there were no differences in the total FSFI score, the domain scores and the frequency of FSD between middle-aged Korean women with subclinical thyroid hypofunction and their peers with normal thyroid status [7]. In a Chinese study, FSD, hypolibidemia, low arousal, impaired lubrication, low orgasm, low sexual satisfaction and sexual pain were not more frequent in women with subclinical hypothyroidism than in controls with normal thyroid function, but most cases (86%) of hypothyroidism were of non-autoimmune origin [8]. Lastly, a meta-analysis including the results of seven studies showed that the presence of overt hypothyroidism had an unfavorable effect on lubrication, and, though to a lesser degree, on the remaining domains of the FSFI. In turn, the impact of subclinical hypothyroidism on female sexual response was limited to a decrease in sexual arousal and orgasm [9].

Postpartum thyroiditis (PPT) is an autoimmune thyroid disease occurring in the first year after delivery and, rarely, after a miscarriage, and is caused by the immunological rebound that follows the partial immunosuppression of pregnancy [10,11]. The clinical presentation of PPT is variable because patients may develop a transient thyrotoxic phase followed by a transient phase of thyroid hypofunction, isolated thyrotoxicosis and isolated hypothyroidism [12]. The thyrotoxic phase of PPT is usually short in duration (2–6 weeks), and often goes unnoticed before a longer-lasting hypothyroid phase or permanent hypothyroidism develops [12,13]. Like Hashimoto’s thyroiditis, PPT is characterized by the presence of specific antibodies and a lymphocytic infiltration of the thyroid gland, but without germinal centers, oxyphil cell metaplasia and glandular fibrosis [14]. The incidence of PPT in the general population is estimated to be between 5 and 8%, but due to the insufficient awareness of this clinical entity, most cases are unrecognized [11,15].

Despite many similarities of PPT and Hashimoto’s thyroiditis and the development of transient or permanent hypothyroidism in these disorders, no study investigated female sexual response in patients with the former disease. Interestingly, impaired sexual health in women was found to be accompanied by depressive symptoms [3,5], which are often reported in the postpartum period [16,17]. Although studies evaluating the relationship between PPT and depression provided inconsistent results [18], thyroid autoimmunity in the postpartum period was found to predispose to depressive symptoms [19,20]. Thus, the aim of our study was to investigate whether PPT has an impact on female sexual function and depressive symptoms.

## 2. Materials and Methods

All patients signed the written informed consent after receiving thorough information about the study. The protocol was approved by the appropriate ethical committees. All procedures complied with the 1964 Declaration of Helsinki and its subsequent revisions.

### 2.1. Study Population

This study included women (aged 18–45 years) with recently diagnosed and untreated PPT. The participants were recruited during 2015–2018 by the principal investigator among white Polish women inhabiting the Częstochowa district of the Silesian Voivodeship, which is the largest urban area in Poland. To be admitted to the study, they were required to have (a) positive thyroid peroxidase antibodies (TPOAb) (titers above 100 U/mL); (b) multifocal areas of reduced echogenicity scattered throughout both lobes of the thyroid gland resulting in a heterogeneous appearance, but without atrophy and fibrosis; and (c) the lack of clinical, laboratory or sonographic signs of Hashimoto’s thyroiditis and other thyroid disorders in the last 12 months preceding pregnancy. Only women who had stopped breastfeeding at least 3 months before the study and who were euthyroid 6 years after the study were considered eligible for inclusion. This study included only women with positive partner relationships in the last 12 months. Partner relationships were assessed by asking about the most important aspects of partner relations (love, investment and the lack of conflicts). Both potential participants and their partners were separately questioned, and the answers had to be consistent. Potential participants were also excluded if they met at least one of the following criteria: the thyrotoxic phase of PPT, another chronic disorder, any acute disease, psychiatric problems, poor self-reported psychological well-being (defined based on the criteria proposed by Herzlich [21]), sexual inactivity, bisexual orientation, pregnancy, previous urogynecological operations that might affect sexual function, developmental or acquired anomalies of the reproductive system or any treatment within the last 8 weeks. Based on TSH and thyroid hormone levels, the participants were assigned to one of three groups: women with overt hypothyroidism (group A), women with subclinical hypothyroidism (group B) and women with euthyroid PPT (group C). The control group consisted of healthy women who gave birth within the last 12 months and were matched with the remaining study groups for age, body mass index and blood pressure. Thyroid function was considered normal if TSH and thyroid hormone levels were within a normal range (TSH between 0.4 and 4.5 mU/L, free thyroxine between 11.0 and 21.5 pmol/L and free triiodothyronine between 2.5 and 6.7 pmol/L). Overt hypothyroidism was diagnosed if TSH levels were higher than 10.0 mIU/L and free thyroxine levels were below the lower limit of the reference range. Subclinical hypothyroidism was defined as elevated TSH (above 4.5 mU/L) with normal free thyroxine and free triiodothyronine concentrations. A sample size calculation showed that 31 patients per group were needed to detect a 15% difference between the study populations in the total FSFI score (the primary endpoint) with a power (β) of 0.9 and a confidence (α) of 0.05. Each group consisted of 40 women, so that the sample size requirement was met. To limit a possible influence of seasonal fluctuations in the outcome variables [22,23,24,25,26], similar numbers of women were assessed in each season (43 between January and March, 39 between April and June, 38 between July and September and 40 between October and December).

### 2.2. Laboratory Assays

Blood samples were collected from the antecubital vein between 7.00 and 8.00 a.m., at least 12 h after the last meal. In menstruating women, samples were taken during the follicular phase. Before venipuncture, all women had been resting for at least 30 min in the seated position. Because of pulsatile secretion and an increase in prolactin secretion induced by stress and phlebotomy [27,28], prolactin levels were assayed in three samples collected at 20 min intervals, and the concentration was averaged. The samples were then coded so that the technicians performing the laboratory assays were blinded to the women’s identity, clinical presentation and results of other objective tests. All measurements were performed in duplicate to ensure data reproducibility, and the average of the two values was used in statistical analyses. Serum titers of TPOAb and thyroglobulin antibodies (TgAb), and levels of TSH, free thyroxine, free triiodothyronine, testosterone, dehydroepiandrosterone sulfate (DHEAS), estradiol and prolactin were assayed by direct chemiluminescence using acridinium ester technology (ADVIA Centaur XP Immunoassay System, Siemens Healthcare Diagnostics, Munich, Germany). The intra-assay and inter-assay coefficients of variations were as follows: 4.0% and 6.3% for TPOAb, 4.3% and 8.0% for TgAb, 2.0% and 3.4% for TSH, 2.8% and 4.8% for free thyroxine, 3.1% and 4.9% for free triiodothyronine, 4.7% and 5.9% for testosterone, 5.1% and 6.4% for DHEA-S, 4.0% and 6.9% for estradiol and 3.1% and 5.0% for prolactin. The assay sensitivities were 8 U/mL for TPOAb, 7 U/mL for TgAb, 0.008 mU/L for TSH, 1.3 pmol/L for free thyroxine, 0.3 pmol/L for free triiodothyronine, 0.2 nmol/L for testosterone, 0.08 μmol/L for DHEA-S, 30 pmol/L and 0.7 ng/mL for prolactin.

### 2.3. Questionnaires

Questionnaires were filled in immediately after venipuncture. The participants answered the questions in a separate room without help or interference from the researchers. During this part of the study, the women and the investigators were unaware of the biochemical test results. Referring to previous literature [29,30], we collected sociodemographic data on patients’ characteristics (including age, smoking status, physical activity level, education, occupational status, sexual partners, marital status, delivery history, miscarriage history and stress exposure).

Sexual functioning was assessed using FSFI, a 19-item questionnaire analyzing six separate domains of the functional aspect of female sexuality over the past 4 weeks: desire (2 items), arousal (4 items), lubrication (4 items), orgasm (3 items), satisfaction (3 items) and penetration pain (3 items) [31]. The questionnaire recognizes the multidimensional nature of female sexual functioning [32]. Responses are graded on a scale from 1 (almost never or never) to 5 (almost always or always), where a score of 0 indicates no sexual activity. By adding the scores of the individual items comprising the domain and by multiplying the sum by the respective domain factor (0.6 for desire, 0.3 for arousal and lubrication and 0.4 for the remaining domains), individual domain scores are calculated [32]. The domain factors depend on the number of questions per domain and are used to equally weigh each domain (higher scores indicate better sexual health). Women having a total score of ≤26.55 are defined as having FSD [31].

The last questionnaire, the Beck Depression Inventory—Second Edition (BDI-II), is a 21-item questionnaire that addresses the most common symptoms of depression [33,34]. The items cover a range of emotional, cognitive, motivational and somatic components of this disorder and are designed to reflect the depressive episode criteria outlined in the DSM-IV [34]. The BDI-II is answered on a scale ranging from 0 to 3, with a final score between 0 and 63 points. A higher score on the BDI-II denotes more severe depression [34]. A cut-off of 0–13 points indicates no/minimal depressive symptoms, 14–19 points indicates mild depressive symptoms, 20–28 points indicates moderate depressive symptoms and 29–63 points indicates severe depressive symptoms [34].

### 2.4. Statistical Analysis

All quantitative variables were log-transformed to ensure normality of the distribution, to stabilize variance and to handle outliers. Between-group comparisons were performed using one-way analysis of covariance followed by Bonferroni’s post hoc tests after consideration of age, body mass index as well as blood pressure as potential confounding factors. Qualitative variables were compared using the chi-square test. Bivariate correlations were determined using Pearson’s r tests (for two quantitative variables), point-biserial (for two qualitative variables) and phi coefficient (for one quantitative and one qualitative variable). To find biochemical variables having a significant effect on sexual functioning and depressive symptoms, multivariate regression analysis was performed with the total FSFI score, FSFI domain scores and the BDI-II score as dependent variables and antibody titers and hormone levels as independent variables. All differences with a *p* value less than 0.05 were considered statistically significant.

## 3. Results

### 3.1. General Characteristics of the Study Groups

The study groups did not differ in age, time since delivery, duration of symptoms, smoking, physical activity, education, occupational activity, type of work, the number of sexual partners, the number and duration of marriages, the number of deliveries and miscarriages, stress exposure, body mass index and blood pressure (Table 1).

### 3.2. Biochemical Variables

Titers of TPOAb and TgAb, and levels of TSH, were higher in group A than in the remaining groups, higher in group B than in groups C and D and, except for TSH, higher in group C than in group D. Free thyroxine and free triiodothyronine were lower in group A than in groups B–D and lower in group B than in groups C and D. Testosterone was lower in groups A and B than in groups C and D, and lower in group C than in group D. DHEAS did not differ between groups A, B and C, but was lower in all groups of women with PPT than in the control group. Estradiol was lower in group A than in the remaining groups. Prolactin was highest in group A, and higher in group B than in groups C and D (Table 2).

### 3.3. Sexual Functioning

The total FSFI score and all domain scores were lower in group A than in the remaining three groups and lower in group B than in groups C and D. Groups C and D differed in the total FSFI score and in domain scores for desire, arousal and lubrication, but not in domain scores for orgasm, sexual satisfaction and pain. FSD was observed in 73% of women in group A, 53% of women in group B, 30% of women in group C and 12% of women in group D (Table 3).

### 3.4. Depressive Symptoms

The overall BDI-II score, and the percentage of patients with total and mild depressive symptoms, differed between group A and groups B–D and between group B and C and group D. The highest values were observed in group A, while the lowest ones in group D. Apart from two patients in group A, one woman in group B and two patients in group C, there were no cases of moderate or severe depressive symptoms in the study population (Table 4).

### 3.5. Correlations

The total FSFI score and all domain scores inversely correlated with TPOAb titers in all groups of women with PPT and positively correlated with free thyroxine and free triiodothyronine levels in groups A and B. Moreover, there were positive correlations between the total FSFI score, and scores for desire and arousal and testosterone in all study groups; inverse correlations between lubrication, orgasm, sexual satisfaction and pain and prolactin in groups A and B; and a positive correlation between lubrication and estradiol in group D (Table 5).

The overall BDI-II score inversely correlated with the total FSFI score and all domain scores (Table 6).

### 3.6. Multivariate Regression Analysis

Multivariate regression analysis showed that TPOAb and free thyroxine were independent and significant determinants of sexual functioning in hypothyroid women with PPT (partial R^2^ between 0.248 [*p* = 0.0015] and 0.328 [*p* < 0.0001] for TPOAb; partial R^2^ between 0.211 [*p* = 0.0028] and 0.308 [*p* < 0.0001] for free thyroxine), while only TPOAb were independently and significantly associated with sexual function in euthyroid women with this disorder (partial R^2^ between 0.287 [*p* = 0.0001] and 0.359 [*p* < 0.0001]). Multivariate regression analysis showed no relationship between the BDI-II score and the investigated biochemical variables.

## 4. Discussion

This study shows for the first time that PPT is complicated by impaired sexual functioning, and that women with the highest antibody titers and the lowest thyroid hormone levels in the first postpartum year are particularly predisposed to the development of FSD. The study design, particularly the long period (6 years) between the study and detecting normal thyroid function, ruled out a possibility of overlapping effects of postpartum thyroid dysfunction and permanent hypothyroidism that was diagnosed for the first time several months after delivery. The obtained results cannot be explained by the impact of age, body mass and blood pressure. Although all these factors were found to affect women’s sexual behavior [35,36,37], while hypothyroidism additionally predisposes to increased weight and hypertension [38], the study groups were matched for age, body mass index and blood pressure, excluding such a possibility. They cannot be also attributed to unfavorable effects of comorbidities or pharmacotherapy because our patients did not have concomitant disorders and were untreated. Similarly, because of the lack of between-group differences, our findings seem not to be associated with differences in time since delivery, duration of clinical symptoms, smoking habits, physical activity, education, occupational activity, stress exposure, previous sexual life and earlier pregnancies. Lastly, the subjects under study were unaware of the thyroid testing, unlike previous studies, in which patients’ knowledge of their own thyroid status was an important confounding factor. Thus, multidirectional disturbances in sexual functioning seem to be a specific, although not previously reported, manifestation of PPT.

The current study provides some conclusions concerning sexual functioning in hypothyroid women with PPT. Firstly, the degree of its impairment is determined by the severity of hypothyroidism, and is more pronounced in the case of overt than subclinical thyroid hypothyroidism. Secondly, except for decreased mood, female sexual response was unrelated to the remaining clinical symptoms of thyroid hypofunction. Thirdly, impaired sexual function is partially a consequence of action of low levels of thyroid hormones at the level of target tissues, which is supported by positive correlations between the overall and all domain scores and serum concentrations of free thyroid hormones, and by an independent association between sexual function and free thyroxine in women with hypothyroidism. Fourthly, the lack of correlations argues against the direct involvement of TSH. This finding is in line with the hypothesis that TSH secretion is influenced mainly by local type 2 deiodinase, making pituitary cells more sensitive to thyroid hormones in comparison to the remaining tissues, and that circulating TSH levels may not accurately reflect thyroid hormone action at the peripheral level [39].

Though less pronounced than in hypothyroid patients, disturbances in sexual function were also observed in women with PPT and levels of TSH and free thyroid hormones within the reference range. Interestingly, sexual functioning in this group of patients partially differed from that reported by euthyroid women with Hashimoto’s thyroiditis, participating in our previous study [3]. Although both disorders were accompanied by impaired libido and lubrication, only PPT was characterized by impaired arousal, while only Hashimoto’s thyroiditis was associated with reduced sexual satisfaction. FSD in euthyroid women with PPT was even more spectacular in comparison to women enrolled by other research groups. Bortun et al. [4] and Pasquali et al. [6] did not observe significant differences in sexual functioning between reproductive-aged women with euthyroid Hashimoto’s thyroiditis and control subjects [4]. In turn, Oppo et al. [40] reported that hypothyroid patients with Hashimoto’s thyroiditis were characterized by reduced desire, satisfaction and pain before (but not after) normalization of TSH levels, which suggested their association with thyroid hypofunction rather than with thyroid autoimmunity. Because these studies included women at least 12 months after delivery, individuals with postpartum thyroid dysfunction were not enrolled. The obtained results allow us to hypothesize that women with PPT are characterized by impaired sexual function even if the activity of the hypothalamic–pituitary–thyroid axis is within the reference range. Moreover, the severity of FSD in PPT seems to be similar or greater than in Hashimoto’s thyroiditis. Referring our findings to the results of previously published studies, we would like to emphasize three issues. Firstly, our study population exclusively included women with both thyroid antibodies exceeding the reference range and characteristic sonographic imaging findings, while other researchers diagnosed Hashimoto’s thyroiditis based on only positive thyroid antibodies [4,6,40]. Thus, infiltration of the thyroid gland with inflammatory cells was probably more pronounced in our study, and our population was characterized by a lower risk of misdiagnosis than women participating in other studies. Secondly, FSD was observed although thyroid autoimmunity and hypofunction spontaneously resolved later in all participants, while Hashimoto’s thyroiditis is an irreversible disorder [15]. Lastly, our findings indicate that sexual functioning differs between women with various autoimmune disorders of the thyroid gland.

Correlations between sexual functioning and TPOAb titers and the results of multivariate regression analysis consistently indicate that FSFI is determined by severity of PPT. The lack of similar correlations between sexual functioning and TgAb titers may be explained by the fact that TgAb are less sensitive and specific than TPOAb are, and may be negative even if TPOAb titers are markedly elevated [41]. There are at least three possible explanations for the association between PPT and FSD. Firstly, impaired sexual response may be a consequence of low thyroid hormone levels in target tissues. A discrete reduction in peripheral thyroid hormone availability may be observed even in subjects with circulating levels of TSH and free thyroid hormones within the reference range [38,39]. Secondly, PPT is often accompanied by other autoimmune disorders including systemic lupus erythematosus and rheumatoid arthritis, and the course of these disorders may be asymptomatic or oligosymptomatic. These disorders, already at an early stage, have an unfavorable impact on female sexual response [42,43]. Lastly, as the obtained results show, PPT may be associated with secondary changes in the production, and/or metabolism of hormones playing a role in the regulation of sexual response.

In line with the last hypothesis, impaired sexual functioning correlated with testosterone and prolactin concentrations, which differed between the study groups. Testosterone was lower in women with PPT than in their peers with normal thyroid function, in hypothyroid patients than in euthyroid patients and in women with overt than in women with subclinical thyroid hypofunction. In turn, prolactin levels were higher in women with than without thyroid hypofunction, and this difference was particularly pronounced in the overt disease, which is in agreement with the commonly accepted view that hypothyroidism is one of the causative factors for prolactin excess [27]. Importantly, only TPOAb titers and low free thyroxine levels, but not testosterone and prolactin concentrations, were independently associated with impaired sexual functioning. This finding suggests that between-group differences in both hormones are secondary to thyroid autoimmunity and/or thyroid hypofunction, characterizing PPT. The involvement of testosterone and prolactin in mediating the unfavorable impact of PPT on female sexual function has theoretical justification and is supported by our findings. Ovarian and adrenal production of testosterone was found to be inhibited by the proinflammatory state [44], characterizing PPT [12]. Sexual drive and arousal in women with PPT were proven to be under control of testosterone to a greater degree than the remaining aspects of female sexual functioning [45]. Libido and excitement were also the only domains of the female sexual cycle correlating in our study with testosterone concentration. In turn, the remaining domains—lubrication, orgasm, sexual satisfaction and pain—inversely correlated with prolactin levels. Unlike women with primary hyperprolactinemia [46,47], there were no correlations between prolactin concentration and libido and arousal, which is in contrast with disturbances in all aspects of female sexual response observed in women with primary hyperprolactinemia [46,47]. These conflicting results are likely to be a consequence of lower prolactin levels, even in women with the overt disease, than in those participating in the earlier studies [46,47]. Alternatively, other mechanisms mediating the effect on women’s sexual health may partially mask the impact of prolactin excess. Unlike testosterone and prolactin, the effect on sexual functioning does not seem to be mediated by alterations in estradiol levels. Although these levels were reduced in women with overt thyroid hypofunction, the only correlation of estradiol was a weak correlation with lubrication in control subjects.

Another principal finding of the current study was the presence of between-group differences in mood. The decrease in the BDI-II score and an increase in the percentage of patients with total and mild depressive symptoms were noticeable in all groups of women with PPT, though the differences in comparison to healthy subjects were particularly pronounced in case of individuals with overt hypothyroidism. These findings cannot be explained by sociodemographic characteristics of the investigated groups of patients. Interestingly, we have observed that depressive symptoms inversely correlated with sexual functioning. Although this finding suggests that both these effects were mutually related, the causal relationship remains unclear. Thus, longitudinal or interventional studies are required to determine whether FSD leads to depression or vice versa. Some arguments may, however, indirectly suggest that disturbances in female sexual response contribute to reduced mood. Firstly, the overall BDI-II score correlated with all dimensions of female sexual response, providing evidence on the association with not only psychological but only physical aspects of sexual health. Secondly, unlike correlations with sexual functioning, the indices of thyroid autoimmunity, activity of the hypothalamic–pituitary–thyroid axis and levels of the remaining hormones did not correlate with the total BDI-II score and the percentage of patients with depressive symptoms. Thus, between-group differences in mood do not seem to be a direct consequence of thyroid autoimmune disease and/or hypothyroidism. Certainly, it cannot be completely excluded that mood is regulated mainly by locally produced triiodothyronine, and its subnormal brain content may not be accurately reflected by their serum levels. Although the underlying mechanism was not investigated, the association between sexual disturbances and depressive symptoms in PPT may be partially explained by overproduction of proinflammatory cytokines and/or low serotonin content. Both these putative pathophysiological mechanisms of PPT [48,49] seem to play a role in the development of FSD [50,51] and affective disorders [52,53]. However, because the reported correlations were moderate, other factors not assessed in the current study may also contribute to impaired mood in women with PPT.

There are also other conclusions that can be drawn from our findings. Firstly, between-group differences in testosterone, DHEAS, prolactin and estradiol seem to indicate that hormonal dysfunction in PPT is not limited to abnormal functioning of the hypothalamic–pituitary–thyroid axis, but is accompanied by altered secretory activity of other endocrine organs. Secondly, unlike some endocrine and metabolic conditions (macroprolactinemia [46] and vitamin D insufficiency [29]), there is no one specific domain of the FSFI questionnaire that is particularly prone to be affected by PPT. Thirdly, women with FSD developing in the first year after delivery should be investigated for the presence of PPT, even if TSH and thyroid hormone levels are within the reference range. Fourthly, already mild thyroid hypofunction induced by PPT may be complicated by multidirectional disturbances in sexual functioning. Fifthly, because of the relatively short duration of PPT, impaired sexual functioning does not seem to be associated with disturbances in vascular supply and neurogenic regulation, playing a role in the pathogenesis of FSD associated with chronic disorders [54]. Lastly, it seems reasonable to assume that only TPOAb titers predict the risk of FSD in PPT, and additional assessment of TgAb does not offer any extra benefits.

According to the current recommendations, levothyroxine treatment of PPT is not obligatory. The treatment should be started only in women with significant symptoms, women who are actively attempting pregnancy and those currently lactating, and discontinuation of therapy should be attempted after 12 months (except women planning pregnancy or pregnant) [18]. Our study was a case-control observational study, and we did not investigate the impact of thyroid hormone substitution. We are currently performing a study comparing female sexual response in levothyroxine-naïve and levothyroxine-treated women with this disorder (Krysiak et al., unpublished). Theoretically, women with PPT and FSFI may benefit from treatment with levothyroxine and/or non-hormonal agents decreasing TPOAb titers. Unfortunately, studies investigating sexual response in levothyroxine-treated women included only women with hypothyroidism and Hashimoto’s thyroiditis (but not with PPT), providing inconsistent results. Unlike multi-domain benefits observed by Oppo et al. in women with autoimmune hypothyroidism [40], individuals with borderline high–average TSH levels receiving this hormone were characterized by impaired functioning in all domains of the FSFI questionnaire [55], while levothyroxine-treated women with TSH levels within normal limits more frequently developed FSD, and scored significantly lower in desire, arousal and penetration pain than women with no history of thyroid hypofunction [56]. It is, however, difficult to conclude based on the effects of levothyroxine in a completely different disease, in which thyroid hormone substation is permanent [39]. An alternative treatment option for euthyroid women with PPT may be non-hormonal agents reducing thyroid antibody titers, such as vitamin D, myo-inositol or selenomethionine. All of these increased the overall FSFI score and domain scores for desire, arousal, lubrication and sexual satisfaction in women with euthyroid Hashimoto’s thyroiditis, but the effect of vitamin D was more pronounced than that of the remaining two agents [57].

There are some important limitations to this study that should be considered when interpreting our findings. Although the current sample size meets statistical requirements, the inclusion of only 40 patients per group may limit the generalizability of the findings. Expanding the sample size would enhance statistical power. Self-reported measures might have been influenced by human error, subjectivity or intentional misrepresentation. No questionnaire assessment of partner relationships and psychological well-being may be a potential confounding factor influencing sexual function and depressive symptoms. It is hard to extrapolate our findings to breastfeeding women with PPT, not participating in the study. Owing to obligatory iodine prophylaxis and low selenium intake by subjects inhabiting the Upper Silesia [58,59], the impact of PPT on sexual functioning and depressive symptoms does not have to be the same in iodine-depleted and selenium-sufficient areas. The study design does not allow us to draw conclusions about sexual function and mood in the thyrotoxicosis phase of PPT and in postpartum Graves disease. Because questionnaires were completed only once, it cannot be ruled out that female sexual response in women with PPT changes with time. This study does not provide insight into molecular aspects of the association between PPT and female sexual response. Lastly, female sexual response and mood in PPT may be theoretically affected by pharmacological treatment.

## 5. Conclusions

PPT is complicated by multidimensional impairment of female sexual functioning, which is observed even in euthyroid women with this disorder. The unfavorable effect on all aspects of sexual response is potentiated by the resultant hypothyroidism, and correlates with thyroid hormone levels. In addition to thyroid autoimmunity and hypofunction of the hypothalamic–pituitary–thyroid axis, impaired sexual functioning may be partially explained by a decrease in testosterone and an increase in prolactin levels. Low sexual health is linked to depressive symptoms, observed in all groups of women with PPT. These novel findings, expanding current knowledge about clinical manifestations of PPT, require confirmation in larger cohorts.

The following clinical recommendations can be drawn from this study. Firstly, women with PPT are at high risk of FSD and mood worsening. Therefore, we recommend assessment of sexual health and depressive symptoms in all women with this disorder. Secondly, FSD developing during the first 12 months after delivery may suggest the presence of PPT. Therefore, assessment of at least TSH and TPOAb should be mandatory for all women complaining of impaired sexual functioning in the postpartum period. Thirdly, the risk of FSD and of its severe course are particularly high in patients with the most severe cases of this disorder. Fourthly, our findings emphasize the association between sexual functioning and mood in PPT. Hence, we recommend assessment of depressive symptoms in all women with PPT and FSD, and assessment of sexual functioning in all women with depressive symptoms developing in the first postpartum year. Lastly, we recommend further studies aimed at evaluating whether sexual functioning and depressive symptoms improve after either spontaneous normalization of thyroid function or after treatment with levothyroxine and non-hormonal agents reducing thyroid autoimmunity.

## Figures and Tables

**Table 1:** General characteristics of the study groups.

Variable	Group A	Group B	Group C	Group D
**Number of patients**	40	40	40	40
**Age** (years)	32 ± 6	30 ± 5	31 ± 5	31 ± 6
**Time since delivery** (weeks)	31 ± 10	32 ± 11	30 ± 12	31 ± 11
**Duration of symptoms** (weeks)	6 ± 4	7 ± 4	6 ± 3	-
**Smokers** (%)/**Number of cigarettes a day** (n) **/Duration of smoking** (months)	25/12 ± 5/73 ± 24	23/11 ± 5/76 ± 26	23/11 ± 6/78 ± 30	27/10 ± 6/79 ± 32
**Physical activity: total/several times a week/once a week/once a month** (%)	57/20/20/17	57/20/25/12	60/23/25/12	65/25/25/15
**Primary or vocational/secondary/university education** (%)	30/35/35	27/35/38	35/30/35	30/30/40
**Occupational activity**/**Blue-collar/white-collar/pink-collar workers** (%)	67/27/23/17	65/25/23/17	65/25/25/15	67/25/30/12
**Number of sexual partners** (n)	1.8 ± 0.7	1.8 ± 0.8	1.9 ± 0.7	2.0 ± 0.7
**Number of marriages** (n)/**duration of marriages** (months)	1.2 ± 0.6/58 ± 18	1.1 ± 0.5/51 ± 22	1.1 ± 0.6/52 ± 13	1.2 ± 0.7/56 ± 21
**Number of deliveries** (n)/**Number of miscarriages** (n)	1.1 ± 0.6/0.8 ± 0.5	1.2 ± 0.6/0.8 ± 0.4	1.1 ± 0.5/0.5 ± 0.4	1.2 ± 0.6/0.6 ± 0.5
**Stress exposure** (%)	85	85	88	88
**Body mass index** (kg/m^2^)	26.4 ± 3.8	26.0 ± 3.5	24.8 ± 3.4	24.6 ± 4.5
**Systolic blood pressure** (mm Hg)	130 ± 12	129 ± 12	125 ± 15	125 ± 14
**Diastolic blood pressure** (mm Hg)	76 ± 6	75 ± 6	76 ± 6	75 ± 6

Unlike otherwise stated, the data have been shown as the mean ± standard deviation. Group A: women with PPT and overt hypothyroidism; Group B: women with PPT and subclinical hypothyroidism; Group C: euthyroid women with PPT; Group D: healthy women without thyroid disease (control group).

**Table 2:** Biochemical characteristics of the study groups.

Variable	Group A	Group B	Group C	Group D
**TPOAb** (IU/mL)	1725 ± 695 ^a,b,c^	885 ± 402 ^b,c^	439 ± 195 ^c^	15 ± 12
**TgAb** (IU/mL)	1525 ± 458 ^a,b,c^	1005 ± 475 ^b,c^	546 ± 298 ^c^	18 ± 13
**TSH** (mIU/L)	18.4 ± 4.6 ^a,b,c^	7.0 ± 2.2 ^b,c^	1.8 ± 1.3	1.3 ± 1.0
**Free thyroxine** (pmol/L)	7.6 ± 1.1 ^a,b,c^	14.0 ± 2.0 ^b,c^	15.8 ± 2.4	16.4 ± 2.5
**Free triiodothyronine** (pmol/L)	2.3 ± 0.4 ^a,b,c^	2.9 ± 1.2 ^b,c^	3.5 ± 1.0	3.9 ± 1.4
**Testosterone** (nmol/L)	1.02 ± 0.35 ^b,c^	1.07 ± 0.32 ^b,c^	1.30 ± 0.29 ^c^	1.56 ± 4.0
**DHEAS** (μmol/L)	4.75 ± 0.95 ^c^	4.97 ± 0.85 ^c^	5.10 ± 0.98 ^c^	6.10 ± 1.60
**Estradiol** (pmol/L)	190 ± 80 ^a,b,c^	271 ± 85	285 ± 92	293 ± 101
**Prolactin** (ng/mL)	39.9 ± 13.9 ^a,b,c^	28.9 ± 10.3 ^c^	16.0 ± 6.9	12.2 ± 6.1

The data have been shown as the mean ± standard deviation. ^a^ *p* < 0.05 vs. group B; ^b^ *p* < 0.05 vs. group C; ^c^ *p* < 0.05 vs. group D. Group A: women with PPT and overt hypothyroidism; Group B: women with PPT and subclinical hypothyroidism; Group C: euthyroid women with PPT; Group D: healthy women without thyroid disease (control group). *Abbreviations: DHEA: dehydroepiandrosterone sulfate; TgAb: thyroglobulin antibodies; TPOAb: thyroid peroxidase antibodies; TSH; thyroid-stimulating hormone.*

**Table 3:** Sexual functioning in the study groups.

Variable	Group A	Group B	Group C	Group D
**FSFI score**	22.74 ± 4.12 ^a,b,c^	25.71 ± 3.84 ^b,c^	29.67 ± 4.00 ^c^	32.15 ± 2.98
**FSFI score ≤ 26.55** (n (%))	29 (73) ^a,b,c^	21 (53) ^b,c^	12 (30) ^c^	5 (12)
**Sexual desire**	3.60 ± 1.15 ^a,b,c^	4.18 ± 0.76 ^b,c^	4.85 ± 0.78 ^c^	5.48 ± 0.35
**Sexual arousal**	3.76 ± 1.08 ^a,b,c^	4.28 ± 0.82 ^b,c^	4.73 ± 0.88 ^c^	5.40 ± 0.38
**Lubrication**	4.02 ± 0.75 ^a,b,c^	4.40 ± 0.83 ^b,c^	4.82 ± 0.72 ^c^	5.28 ± 0.46
**Orgasm**	3.28 ± 1.02 ^a,b,c^	3.80 ± 1.04 ^b,c^	4.88 ± 0.85	5.18 ± 0.51
**Sexual satisfaction**	4.01 ± 0.85 ^a,b,c^	4.50 ± 0.73 ^b,c^	5.12 ± 0.73	5.38 ± 0.46
**Pain**	4.07 ± 0.69 ^a,b,c^	4.55 ± 0.78 ^b,c^	5.27 ± 0.51	5.43 ± 0.41

Unless otherwise stated, the data have been shown as the mean ± standard deviation. ^a^ *p* < 0.05 vs. group B; ^b^ *p* < 0.05 vs. group C; ^c^ *p* < 0.05 vs. group D. Group A: women with PPT and overt hypothyroidism; Group B: women with PPT and subclinical hypothyroidism; Group C: euthyroid women with PPT; Group D: healthy women without thyroid disease (control group). *Abbreviation: FSFI: Female Sexual Function Index.*

**Table 4:** Depressive symptoms in the study groups.

Variable	Group A	Group B	Group C	Group D
**BDI-II score** (mean ± standard deviation)	15.6 ± 3.2 ^a,b,c^	12.0 ± 3.4 ^c^	11.6 ± 3.6 ^c^	7.8 ± 3.2
**Depressive symptoms** (n (%))	26 (65) ^a,b,c^	16 (40) ^c^	16 (30) ^c^	5 (13)
**Mild symptoms** (n (%))	24 (60) ^a,b,c^	15 (38) ^c^	14 (35) ^c^	5 (13)
**Moderate symptoms** (n (%))	2 (5)	1 (2)	2 (5)	0 (0)
**Severe symptoms** (n (%))	0 (0)	0 (0)	0 (0)	0 (0)

^a^ *p* < 0.05 vs. group B; ^b^ *p* < 0.05 vs. group C; ^c^ *p* < 0.05 vs. group D. Group A: women with PPT and overt hypothyroidism; Group B: women with PPT and subclinical hypothyroidism; Group C: euthyroid women with PPT; Group D: healthy women without thyroid disease (control group). *Abbreviation: BDI-II: Beck Depression Inventory-Second Edition.*

**Table 5:** Correlations between sexual functioning and the investigated biochemical variables in the study population.

Correlated Variables		Group A	Group B	Group C	Group D
Total FSFI score	TPOAb	−0.382 ^b^	−0.378 ^b^	−0.406 ^b^	0.026
Sexual desire	TPOAb	−0.314 ^a^	−0.325 ^a^	−0.342 ^a^	0.064
Sexual arousal	TPOAb	−0.324 ^a^	−0.367 ^a^	−0.356 ^a^	0.112
Lubrication	TPOAb	−0.290 ^a^	−0.302 ^a^	−0.316 ^a^	−0.043
Orgasm	TPOAb	−0.262 ^a^	−0.286 ^a^	−0.258 ^a^	0.120
Sexual satisfaction	TPOAb	−0.315 ^a^	−0.392 ^b^	−0.308 ^b^	0.078
Pain	TPOAb	−0.344 ^a^	−0.358 ^a^	−0.367 ^a^	−0.005
Total FSFI score	Free thyroxine	0.465 ^c^	0.420 ^b^	0.210	0.146
Sexual desire	Free thyroxine	0.488 ^c^	0.473 ^c^	0.204	0.115
Sexual arousal	Free thyroxine	0.365 ^a^	0.424 ^b^	0.187	0.121
Lubrication	Free thyroxine	0.311 ^a^	0.398 ^b^	0.155	0.098
Orgasm	Free thyroxine	0.465 ^c^	0.442 ^b^	0.107	0.068
Sexual satisfaction	Free thyroxine	0.435 ^b^	0.397 ^b^	0.122	0.178
Pain	Free thyroxine	0.410 ^c^	0.371 ^a^	0.104	0.146
Total FSFI score	Free triiodothyronine	0.321 ^a^	0.315 ^a^	0.185	0.105
Sexual desire	Free triiodothyronine	0.305 ^a^	0.340 ^a^	0.134	0.089
Sexual arousal	Free triiodothyronine	0.347 ^a^	0.322 ^a^	0.124	0.065
Lubrication	Free triiodothyronine	0.368 ^a^	0.295 ^a^	0.108	0.032
Orgasm	Free triiodothyronine	0.294 ^a^	0.309 ^a^	0.056	−0.011
Sexual satisfaction	Free triiodothyronine	0.372 ^b^	0.362 ^a^	0.071	−0.112
Pain	Free triiodothyronine	0.355 ^a^	0.311 ^a^	0.100	−0.104
Total FSFI score	Testosterone	0.432 ^b^	0.414 ^b^	0.408 ^b^	0.384 ^b^
Sexual desire	Testosterone	0.475 ^c^	0.442 ^b^	0.435 ^b^	0.411 ^b^
Sexual arousal	Testosterone	0.435 ^b^	0.428 ^b^	0.415 ^b^	0.402 ^b^
Lubrication	Prolactin	0.385 ^b^	0.392 ^b^	0.121	0.042
Orgasm	Prolactin	−0.428 ^b^	−0.398 ^b^	−0.214	−0.198
Sexual satisfaction	Prolactin	−0.295 ^a^	−0.328 ^a^	−0.195	−0.114
Pain	Prolactin	−0.278 ^a^	−0.298 ^a^	−0.183	−0.094
Lubrication	Estradiol	0.121	−0.046	−0.102	0.287 ^a^

The data represent the correlation coefficients (r values). ^a^ *p* < 0.05, ^b^ *p* < 0.01, ^c^ *p* < 0.001. Group A: women with PPT and overt hypothyroidism; Group B: women with PPT and subclinical hypothyroidism; Group C: euthyroid women with PPT; Group D: healthy women without thyroid disease (control group). *Abbreviations: FSFI: Female Sexual Function Index; TPOAb: thyroid peroxidase antibodies.*

**Table 6:** Correlations between sexual functioning and depressive symptoms in the study population.

Correlated Variables		Group A	Group B	Group C	Group D
BDI-II	FSFI	−0.412 ^c^	−0.422 ^c^	−0.372 ^b^	−0.352 ^b^
BDI-II	Sexual desire	−0.425 ^b^	−0.442 ^b^	−0.412 ^b^	−0.372 ^b^
BDI-II	Sexual arousal	−0.455 ^c^	−0.400 ^b^	−0.352 ^a^	−0.350 ^a^
BDI-II	Lubrication	−0.395 ^b^	−0.348 ^a^	−0.322 ^a^	−0.354 ^a^
BDI-II	Orgasm	−0.362 ^a^	−0.398 ^b^	−0.375 ^b^	−0.341 ^a^
BDI-II	Sexual satisfaction	−0.465 ^c^	−0.382 ^b^	−0.394 ^b^	−0.302 ^a^
BDI-II	Pain	−0.341 ^a^	−0.378 ^b^	−0.314 ^a^	−0.298 ^a^

The data represent the correlation coefficients (r values). ^a^ *p* < 0.05, ^b^ *p* < 0.01, ^c^ *p* < 0.001. Group A: women with PPT and overt hypothyroidism; Group B: women with PPT and subclinical hypothyroidism; Group C: euthyroid women with PPT; Group D: healthy women without thyroid disease (control group). *Abbreviations: BDI-II: Beck Depression Inventory-Second Edition; FSFI: Female Sexual Function Index.*

## Data Availability

The data that support the findings of this study are available from the corresponding author upon reasonable request.

## Abbreviations

BDI-II	Beck Depression Inventory-Second Edition
DHEAS	dehydroepiandrosterone sulfate
FSD	female sexual dysfunction
FSFI	Female Sexual Function Index
PPT	postpartum thyroiditis
TgAb	thyroglobulin antibodies
TPOAb	thyroid peroxidase antibodies
TSH	thyroid-stimulating hormone
